# Supplementary figures and images for: Functional ecological traits in young and adult thalli of canopy-forming brown macroalga Gongolaria barbata (Phaeophyta) from a transitional water system
Source: PeerJ. 2024 Sep 12;12:e17959. doi: 10.7717/peerj.17959 (PMC11402337; doi:10.7717/peerj.17959)

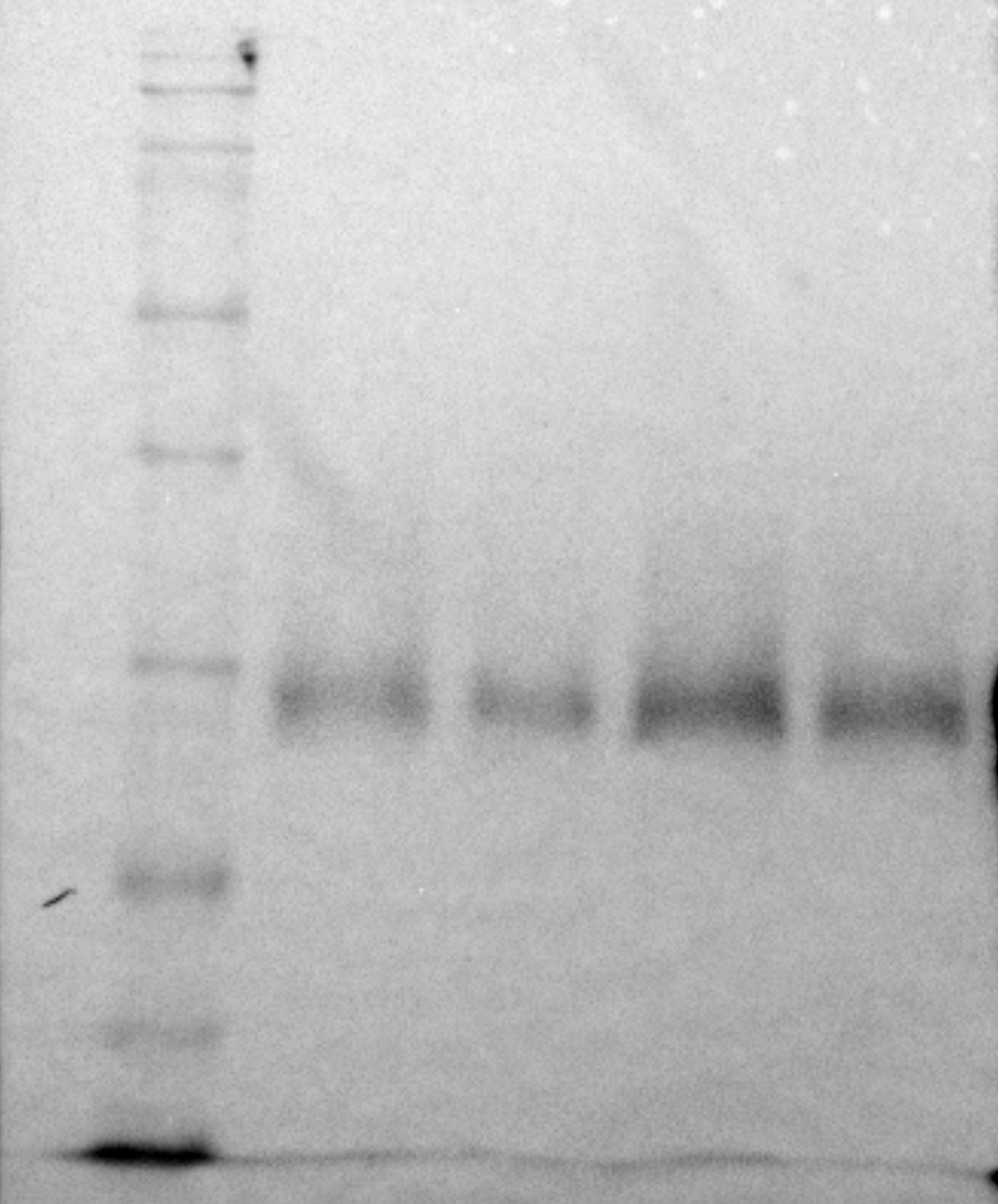

Supplement: Supplemental Information 2 [file peerj-12-17959-s002.png]

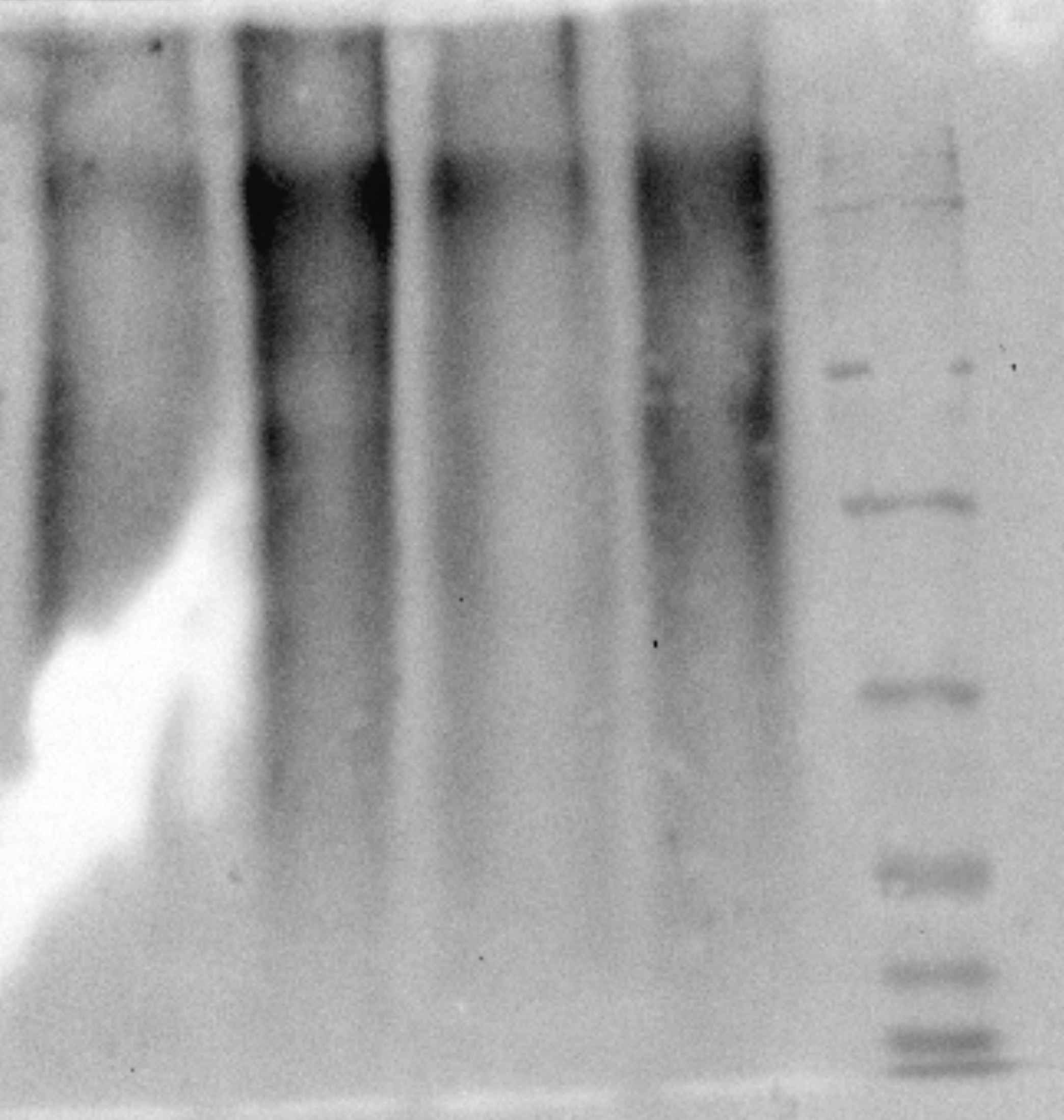

Supplement: Supplemental Information 3 [file peerj-12-17959-s003.png]
